# In Vitro Assessment of the Functional Dynamics of Titanium with Surface Coating of Hydroxyapatite Nanoparticles

**DOI:** 10.3390/ma12050840

**Published:** 2019-03-12

**Authors:** José Henrique de Lima Cavalcanti, Patrícia C. Matos, Cresus Vinícius Depes de Gouvêa, Waldimir Carvalho, José Luis Calvo-Guirado, Juan Manuel Aragoneses, Letícia Pérez-Díaz, Sergio Alexandre Gehrke

**Affiliations:** 1 Department of Rehabilitation, Federal Fluminense University, Rio de Janeiro 20000-000, Brazil; josehenriquecavalcanti@yahoo.com.br (J.H.d.L.C.); cresusuff@gmail.com (C.V.D.d.G.); homensdepalavra@gmail.com (W.C.); 2 Oral Rehabilitation Carioca Centre, Campo Grande 79000-000, Brazil; p.robbs@hotmail.com; 3 Department of Oral Surgery and Implantology, Universidad Católica de Murcia, 30107 Murcia, Spain; jlcalvo@ucam.edu; 4 Department of Dental Research, Universidad Federico Henriquez y Carvajal (UFHEC), Santo Domingo 10107, Dominican Republic; jaragoneses@ufhec.edu.do; 5 Molecular Interactions Laboratory, Facultad de Ciencias, Universidad de la República, Iguá 4225, Montevideo 11400, Uruguay; letperez@gmail.com; 6 Department of Biotechnology, Biotecnos-Technology and Science, Cuareim 1483, Montevideo 11100, Uruguay; 7 Biotechnology Department, Catholic University of Murcia (UCAM), 30107 Murcia, Spain

**Keywords:** implants surface, mesenchymal cells, titanium implants

## Abstract

Manipulation of implant surface characteristics constitutes a promising strategy for improving cell growth and tissue response on a variety of materials with different surface topographies. Mesenchymal progenitor cells with a capacity to respond to titanium surface stimuli and differentiate into osteoblasts were used to perform comparative tests between two different implant topographies, including their functional interaction with pre-osteoblasts directly seeded onto the implants. Functional analysis of nanostructured implant surfaces was performed by in vitro assay analysis. The machined surface of titanium implants (mach group) was used as a control and compared with a nanoparticle HA activated surface implant (nano group), developed by the deposition of pure crystalline hydroxyapatite. Cell culture on the nano group surface resulted in higher cell adhesion and cultured osteoblast viability compared with the mach group. Scanning electron microscope (SEM) images revealed a stable interaction, indicated by the presence of focal cell adhesion formation. These results together with positive mineralization assays showed the nano group to be an excellent scaffold for bone-implant integration.

## 1. Introduction

Currently, titanium implants have a high success rate reported in the world literature, ranging between 90 and 100% [1,2]. However, the search continues to improve the integration of this implant material and maintain the stability of its contact with the surrounding tissues over long periods of functional life. The clinical success of titanium implants is dependent on proper integration with tissues. Moreover, the events derived from the early stages of healing in areas that receive implants represent and determine the future characteristics of the tissues involved in the process. 

In this way, the physicochemical properties of the implant material’s surface are key determinants of biological behavior, as this is the part of the implant that first makes contact with the fluids and cells in the bed where it is inserted. A titanium implant inserted in bone tissue forms an interface with osteoblast cells during the initial stages, when the cells will be mainly dependent on surface topography. In this context, the cell/material interface will itself be dependent on structures and interactions at the nanoscale [3]. 

The in vitro influence of nanostructures on cell activity has been demonstrated with different cell types, indicating that nanoscale surface topography may modulate final tissue formation [4]. This integration occurs through adhesion and cell shape changes. The proliferation and differentiation of osteoblast cells followed by the production of a mineralized matrix can be expected whenever target surfaces offer ideal properties. 

Chemical modifications have also been investigated as a means of modulating tissue reactions to the implant material. In this way, coating the titanium surface with various materials has been proposed but with this technique (coating metals), the micron-thick material may not resist the stress levels resulting from the detachment of coating particles from the implant [5]. Thinner coatings based on nanoparticles, may offer an alternative means of avoiding the sequalae of coat loosening. In recent literature, crystallized hydroxyapatites have been reported to promote cell activity as a stimulator of cell proliferation [6,7] and possibly cell deposition at the level nanoscale. Pure crystalline hydroxyapatite on the titanium surface can improve osseointegration by boosting the osteoinductive potential of the implant surface. 

This work set out to provide important information about implant surface evaluation and to show the promising potential of nanostructured material surfaces to induce cell viability, focal cell adhesion and bone mineralization in vitro. The study investigates these effects comparing a control group of traditional machined titanium surfaces with a test group of nanoparticle HA activated titanium surfaces using pure crystalline hydroxyapatite.

## 2. Materials and Methods

### 2.1. Titanium Implants and Disks

Two different titanium surfaces were evaluated: a machined surface used as control group (mach group), in which the samples did not receive any surface treatment after machining (smooth surface); and an acid-etched conditioning plus deposition of nanoparticle HA-blasted surface (nano group), in which surfaces were modified with hydroxyapatite (HA) using the Promimic nano HA method. The method comprises the formation of a liquid crystalline phase in a water solution of calcium, phosphor and a surfactant, placing the phase in an ammonia atmosphere so that nano-sized crystals are formed. Then, the surfactant is removed with a solvent and the nano-sized crystals recovered to obtain a powder; the ammonia-treated liquid crystalline phase is diluted with a hydrophobic organic solvent to create a microemulsion of nano-sized crystals in water. Then, the oxide layer-coated surface is dipped into the microemulsion or alternatively, the ammonia treatment of the liquid crystalline phase can be postponed until after the surface is dipped into the microemulsion. Finally, the organic solvent and the surfactant are removed from the surface leaving the coating [8]. Samples were briefly dipped into a stable particle suspension containing HA particles of 10 nm diameter followed by heat treatment at 550 °C for 5 min in a nitrogen atmosphere. The surfactant-mediated process allows better control of the coating’s chemical composition [9,10].

Twenty titanium disks and five cylindrical implants in each group were manufactured by S.I.N.—*Sistema Nacional de Implantes* (National Implants System, São Paulo, Brazil). All samples were sterilized by exposure to Gamma irradiation (Embrarrad, São Paulo, Brazil), applying the same care and legal norms required for the commercialization of titanium implants. 

### 2.2. Surfaces Characterization

Five disks in each group were used to characterize the physicochemical composition of the surfaces and determine roughness parameters using scanning electron microscopy (SEM), energy dispersive spectroscopy (EDS), an atomic force microscope (AFM). The surface morphology of the samples in both groups was examined under SEM (JEOL, model JSM 6490-LV, Tokyo, Japan) using the secondary electron (SE) detection mode. For a direct comparison of the surface morphology, the same magnification (1000×) was selected for all samples. The surface chemical composition of all samples was analyzed, using the microscope in EDS mode, in the most central area of each disk; analysis was performed at 200× magnification. Then, the samples were used to generate a series of 3D images using a scanning probe microscope (AFM) (Bruker, Santa Barbara, CA, USA). To measure surface roughness parameters, an optical laser profilometer (Mahr GmbH, Gottingen, Germany) was used, measuring the high variation of the valleys (Z), the absolute values of all profile points (Ra), the root-mean-square of the values of all points (Rq) and the value of the absolute heights of the five highest peaks and the depths of the five deepest valleys (Rz). 

### 2.3. Cell Culture Experiments

MC3T3-E1 (ATCC 7594) murine osteoblastic cells were cultured in α-MEM medium supplemented with 10% fetal bovine serum (FBS) at 37 ℃ in a 5% CO_2_ atmosphere. Confluent cells were trypsinized, diluted and seeded at a cell density of 1 × 10^5^ cells/mL on the indicated surfaces. As a control, cells were cultured in 13-mm Thermanox® coverslips (Thermo Scientific Nunc Inc., Rochester, NY, USA) pre-coated with 0.1% porcine gelatin. Five disks per group were used in each experiment.

### 2.4. Viability Assay

The viability of cultured cells on the two surfaces (match and nano groups) was assessed after 24h through the LIVE/DEAD cell viability assay (ThermoFischer, Waltham, MA, USA). Briefly, the cells were labeled with calcein-AM (AM-Ca) to assess the intracellular esterase activity present in viable cells. Dead cells were labeled using cell-impermeant red-fluorescent ethidium homodimer-1 (EthD-1) as a hallmark of plasma membrane integrity loss in non-viable cells. After incubation for 30 min at 37 ℃ in darkness, cells were washed with PBS for 5 min and images were acquired with an AxioVision 4.8.1 fluorescent microscope (Zeiss, Oberkochen, Germany). The corresponding green (Calcein) and red (EthD-1) fluorescence were detected at 530 and 645 nm respectively using a specific band-pass fluorescence filter. As a positive control, healthy cells were grown on 13-mm Thermanox® coverslips (Thermo Scientific Nunc Inc., Rochester, NY, USA) pre-coated with 0.1% porcine gelatin. As a negative death control, cells were grown on the same surface but incubated with dimethyl sulfoxide (DMSO) instead of α-MEM culture medium. Each surface was tested in five independent experiments and eight representative fields were analyzed at the same magnification for each sample.

### 2.5. Osteoblast Cell Adhesion and Morphology

Adhesion, cell morphology and cell-surface interaction analyses were performed by SEM. MC3T3-E1 cells were seeded at a density of 2 × 10^4^ cells/disc (n = 5 per surface). After 24 h, cells were washed with 0.1 M PBS to remove non-adherent cells, fixed using Karnovsky’s solution (2.5% glutaraldehyde, 4% PFA, 0.1M sodium cacodylate) for 2 h at room temperature, washed three times with 0.2 M sodium cacodylate buffer and post-fixed with osmium tetroxide (1% osmium in cacodylate 1%). Fixed cells were washed with 0.2 M cacodylate and gradually dehydrated adding ethanol/distilled water mixtures containing 30, 50, 70, 90 and 100% volumes of ethanol and critical point drying (BAL-TEC DPC 030) using CO_2_ as ethanol substitute. The samples were metalized with a thin gold film (Emitec, Lohmar, Germany) and analyzed under a SEM microscope (Zeiss).

### 2.6. Focal Contact and Cell-Surface Interaction

For focal adhesion identification, 2 × 10^3^ cells/well were seeded and left for 24 h to adhere to each surface. Cells were fixed in paraformaldehyde 4%, permeabilized with Triton 0.1%, blocked with bovine serum albumin (BSA) 1% and incubated overnight with anti-vinculin antibody diluted 1:150. Cells were PBS washed and incubated with secondary antibody conjugated to Alexa-Fluor 488 (1:500) for 1 h. Fixed cells were also co-incubated with Alexa-Fluor 546 conjugated phalloidin for 30 min (1:50) to stain actin fibers and mounted on glass slides with 4′,6-diamidino-2-phenylindole (DAPI) Fluorshield to stain nucleic acids in the nucleus. Stained cells were digitally registered using the fluorescent filters for vinculin (green), actin fibers (red) and nuclei (blue stains). The Axio Observer microscopy and Z-stack accessories were used to acquire images and AxioVision 4.8.1 (Zeiss, Oberkochen, Germany) software was used for image processing and overlay.

### 2.7. Mineralization Assay

The implants were disposed on specific supports or scaffolds to allow the cellular culture of pre-osteoblasts (MC3T3-E1) on their surfaces. Cells were seeded at a density of 1 × 10^5^ cells/scaffold, kept for 28 days and maintained under cell culture conditions. The α-MEM medium was changed every 3 days and bone mineralization inducers (β-glycerol phosphate, acid ascorbic and melatonin) were added to the positive control. After this period, cells were fixed and the mineralization process was revealed using Alizarin Red S staining solution (40 mM pH 4.2 for 1 h) to detect the calcium matrix (osteogenesis quantitation kit – Millipore, Burlington, VT, USA). Images of the mineralized matrix were acquired by digital microscopy with high resolution LED (KH 7700–Hirox-Europe, Tokyo, Japan). Undifferentiated osteoblasts without extracellular calcium deposits were slightly reddish, whereas mineralized osteoblasts with extracellular calcium deposits resulted in a bright orange-red color. 

## 3. Results

### 3.1. Surface Analysis and Characterization

**Figure 1:**
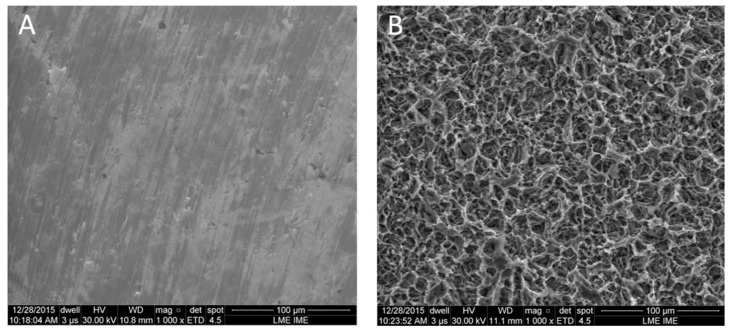
Scanning electron microscopy (SEM) images of the surface characteristics in both groups. (**A**) mach and (**B**) nano groups, respectively.

Analyses of the surfaces’ physical and chemical composition showed obvious differences at the microscopic level between the two surface models analyzed by SEM. The mach group samples were mainly characterized by multidirectional grooves resulting from the machining process (Figure 1A). Samples treated with the nanoparticle HA process showed a homogenous distribution of irregularities over the entire surface and presented ridges and valleys of larger dimensions in many regions, resembling grooves varying from 10 to 20 µm in width (Figure 1B). 

**Figure 2:**
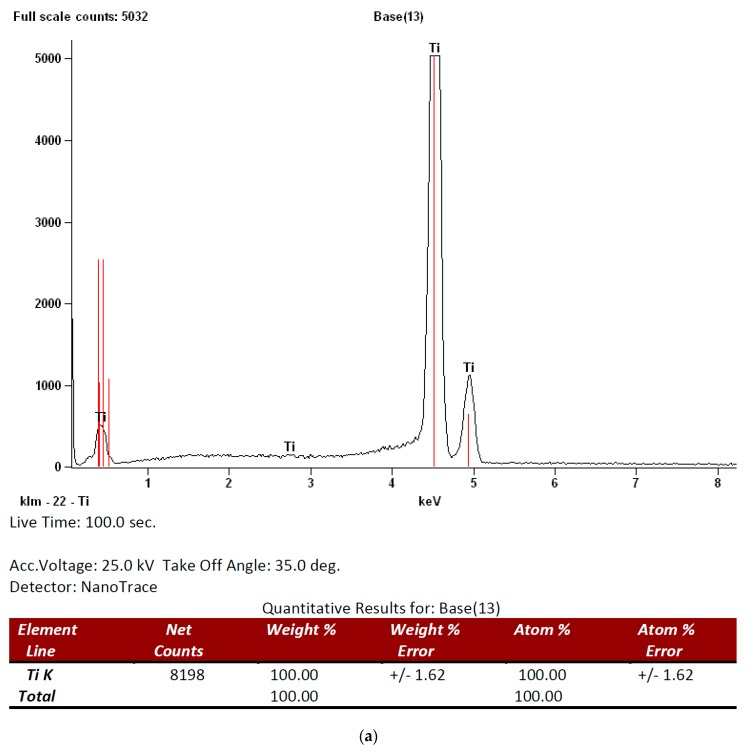

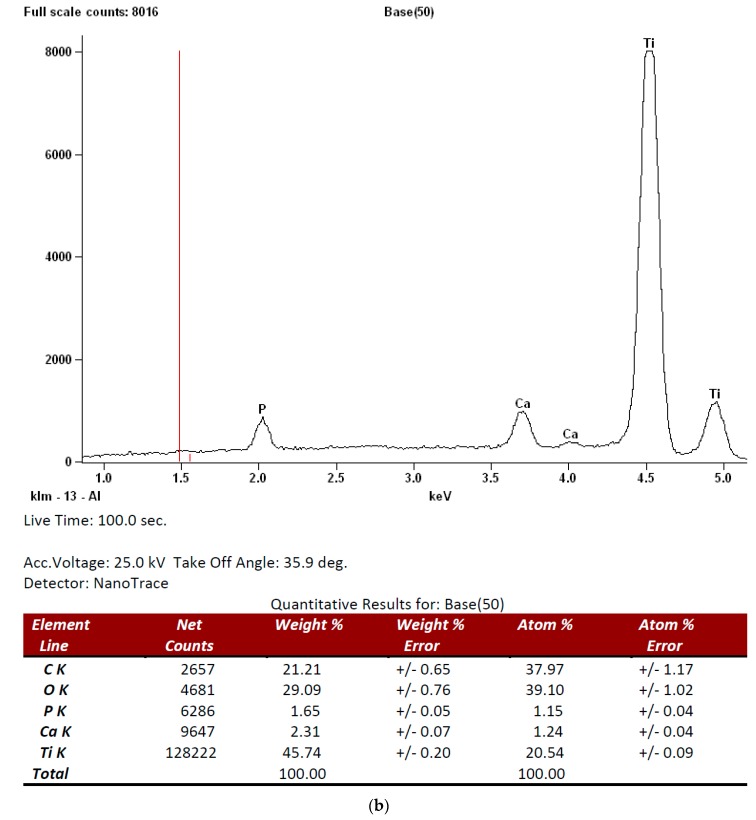
Energy dispersive spectroscopy (EDS) analysis of the surface composition in both groups: (**a**) mach and (**b**) nano groups, respectively).

EDS analysis of the mach group surface found titanium and oxygen to be the only elements in all samples but in the nano group calcium and phosphor were also found on the surfaces in addition to titanium and oxygen. Figure 2 shows the EDS spectrum for both groups.

**Figure 3:**
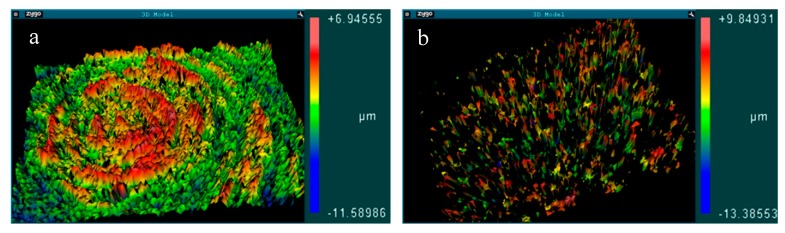
**Atomic force microscopy** (AFM) images of both surfaces analyzed in the study; (**a**) mach and (**b**) nano groups, respectively.

Qualitative and quantitative surface topography analysis observed different degrees of roughness between the two groups. Table 1 shows the mean values of tridimensional roughness parameters determined by profilometry analysis. Surfaces in the mach group showed lower mean values for roughness amplitude parameters than the nano group. The topographic maps obtained by AFM show the qualitative difference in roughness between machined surfaces (Figure 3a) and nanoparticle HA-treated surfaces (Figure 3b). 

### 3.2. Cell Viability Assay of Pre-osteoblast Growing on Different Surfaces

**Figure 4:**
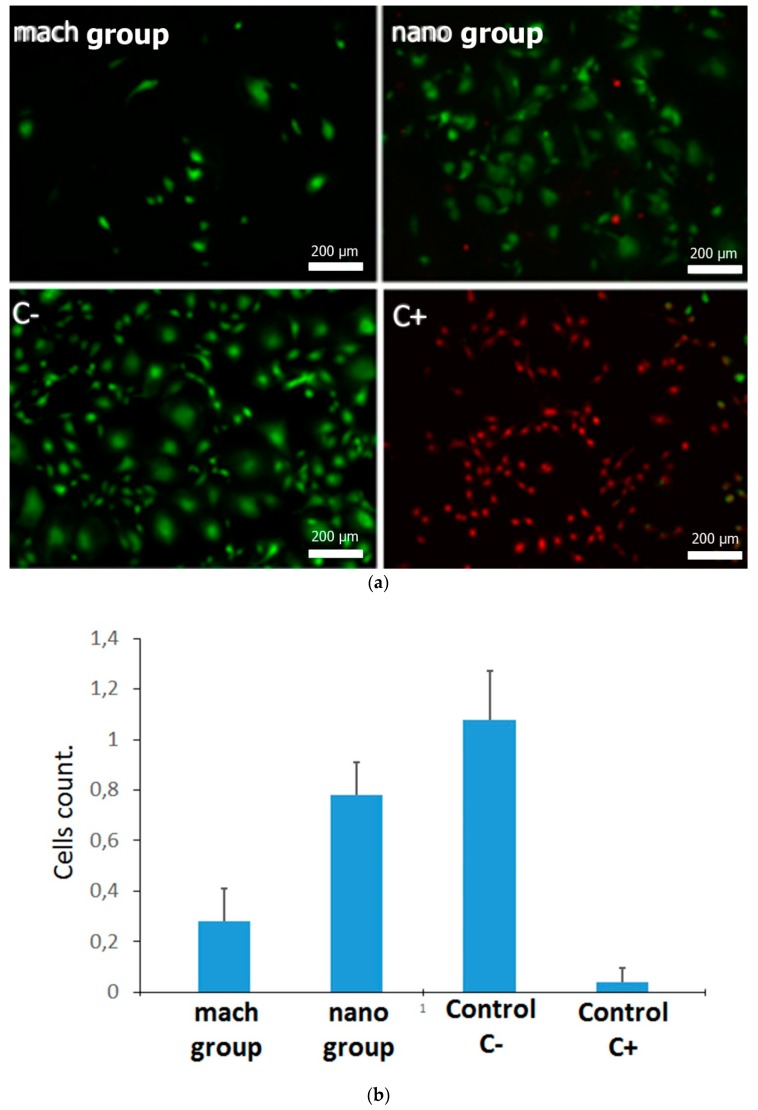
Fluorescence microscopy images of MC3T3-E1 cells adhered to both surfaces of titanium implants ((**a**) mach and (**b**) nano groups) and assay control C− and C+. The mean absorbance values at 570 nm are provided as a box plot. Mean values are expressed as percentages ± Standard Deviation.

In order to compare the selected surfaces as substrates for pre-osteoblast adhesion, MC3T3 cells were seeded on them and cell viability was compared on the two surfaces. Adherent cells were double labeled with Calcein AM and with EthD-1. Calcein AM is a non-fluorescent compound that can easily pass through intact, live cells. Inside the cell, Calcein AM is hydrolyzed by intracellular esterases to Calcein, a fluorescent compound that is well-retained in the cell cytoplasm. EthD-1 can only be internalized in cells whose plasma membrane integrity is compromised and so can be used as a hallmark of non-viable cells. As control of the assay, viable and non-viable cells, MC3T3-E1 cells were cultured on glass coverslips in the presence of culture medium (C−) or in the presence of DMSO (C+) respectively. Viable cells (green) and non-viable cells (red) can be easily distinguished by fluorescence microscopy. The count of viable cells obtained the following means and standard deviations: 0.26 ± 0.12 in the mach group, 0.69 ± 0.27 for nano group, 0.95 ± 0.37 in control C− and 0.04 ± 0.05 in control C+. Although viable cells could be observed on both types of surface (mach and nano groups), the amount of viable adherent cells in the activated nano group surface was notably higher compared with the mach group (Figure 4), with statistically significant differences between the groups (p < 0.0001). This result suggests that the nano group promotes better colonization and distribution of osteoblasts on the material surface than the mach group, a process that is very important during the early stages of osseointegration [8].

### 3.3. Morphological Characterization of Adherent Cells 

**Figure 5:**
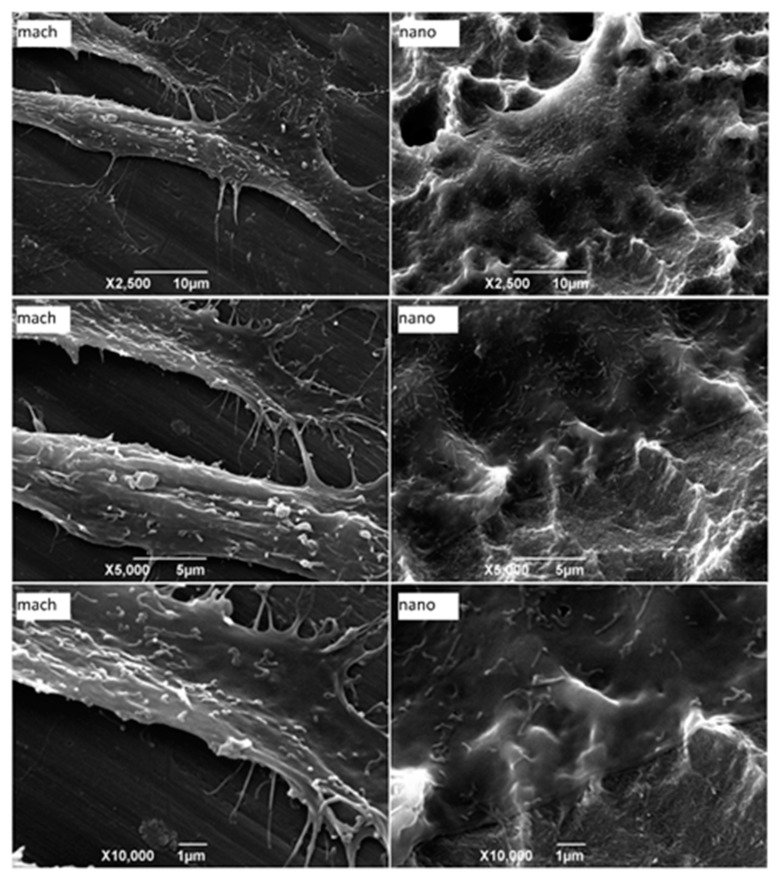
Scanning electron microscopy images of MC3T3-E1 cells adhered to different surfaces of implants (mach and nano groups). After 24 h of cell seeding, the samples were processed for analysis by secondary electron detector. Each image is representative of one of five discs assayed in both groups (2500×, 5000× and 10,000×).

Morphological evaluation of pre-osteoblasts adhered to the substrate can be useful for characterizing cell-surface interaction. To achieve this, pre-osteoblasts were seeded directly onto the mach and nano group surfaces and 24 h later the samples were processed for examination under scanning electron microscopy (SEM). Different magnifications revealed that cell membranes were in close contact with the activated nanoparticle HA surfaces (nano group) and that these accompanied the surfaces’ nanoroughness as a “cover layer.” However, spaces between the plasma membrane and the surface could be seen in cells adhered to the machined surface (mach group), interacting with the substrate through filopodia-like structures (Figure 5). These findings suggest that the interaction of pre-osteoblasts with the nanoparticle HA surface could be more stable since almost the entire cell surface was in contact with the substrate. 

### 3.4. Focal Contact Analysis between Pre-osteoblasts and Implant Surfaces

Focal adhesions are macromolecular structures that mediate cell adhesion. It is here that the closest contact between cells and substrate is formed. Moreover, focal adhesions functionally interact with stress fibers, constituting contractile bundles of actin and myosin. To date, many focal adhesion proteins have been identified in vertebrates including vinculin, talin, paxillin, zyxin, α-actinin, vasodilator-stimulated phosphoprotein (VASP), focal adhesion kinase (FAK), phosphotyrosine proteins, integrin αVβ3 [11] and actopaxin [12]. Vinculin is a cytoplasmic actin-binding protein associated with cell-cell and cell-matrix junctions and so enriched in focal adhesions and adherens junctions [13]. In order to characterize osteoblast attachment to different surfaces, focal adhesions were analyzed through immunofluorescence assay using an antibody against vinculin, a specific focal contact protein and a fluorescent labeled phalloidin, a highly selective peptide used to label actin filaments (also known as F-actin). 

**Figure 6:**
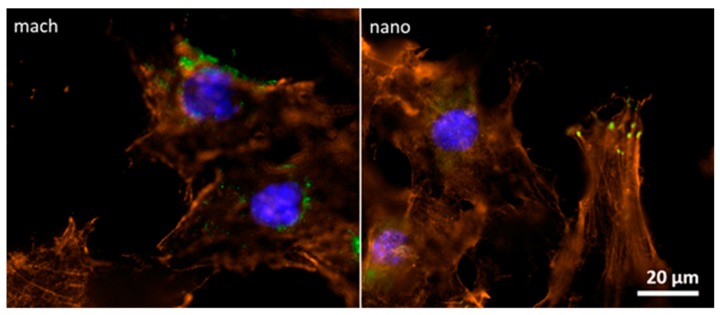
Fluorescence microscopy images of MC3T3-E1 cells adhered to implant surfaces (mach and nano groups). After 24 h cell seeding, samples were fixed and processed for immunofluorescence using an Alexa-Fluor 488 fluorescent antibody to detect vinculin (green), Alexa-Fluor 546 labeled phalloidin to label actin (red) and DAPI to label nucleic acids in the nucleus (blue). Each image is representative of both groups (400×).

The focal adhesion formation for osteoblasts adhered to nano group surfaces was evidenced by the co-localization of actin and vinculin (yellow spots). This focal adhesion was not evidenced in osteoblasts adhered to mach surfaces (Figure 6). This finding concurred with SEM analysis, which observed more contacts between cells and the nanoparticle HA surface. Altogether, these data strongly suggest that the nano group promotes interaction between osteoblasts and the material surface. 

### 3.5. Osteoblast Differentiation on Nano and Mach Groups

Osteoblasts are specialized fibroblasts, derived from mesenchymal precursors, which secrete and mineralize the bone matrix. Their differentiation can be divided into three stages: (a) cell proliferation; (b) matrix maturation; and (c) matrix mineralization [14]. Once mineralization is completed, calcium deposition can be visualized by carrying out staining procedures. In order to functionally evaluate the osteoblast differentiation taking place in the nano and mach groups, young osteoblasts were seeded and maintained under controlled cell culture conditions for 28 days. At the end of this stage, a mineralization test (to determine the success of in vitro bone formation) was performed using calcium staining with Alizarin Red S and analyzed under high resolution LED digital microscopy (Hirox). 

**Figure 7:**
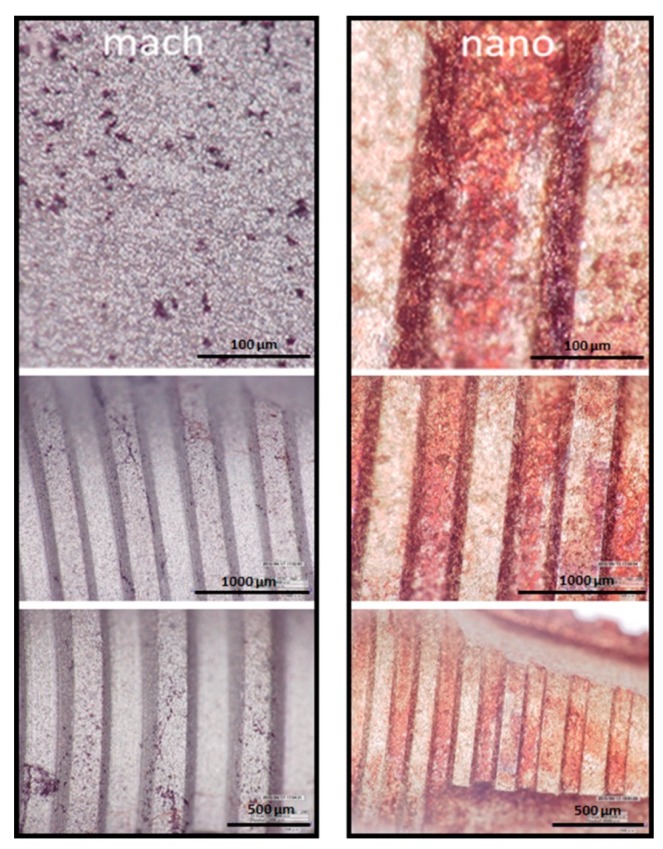
LED digital microscopy images at different resolutions (Hirox) of Alizarin Red S-stained of MC3T3-E1 mineralized cells adhered to implants surface (mach and nano groups, respectively). After 28 days of culture in the presence of osteogenic supplements the cells were processed and stained with Alizarin Red S.

After 28 days cell culture in the presence of osteogenic supplements, the monolayer was more densely stained by orange-red calcium spots, visible to the naked eye, on the nanoparticle HA surface than the machined implant surface (Figure 7). 

Altogether, these two analytic methods revealed higher levels of osteoblast mineralization in cells growing on the nano group samples compared with the mach group.

## 4. Discussion

To verify the biocompatibility of implants and so their long-term performance, biochemical assays at the cellular and molecular level should be performed prior to any clinical trial. In this context, the present work characterized two different titanium implant surfaces: machined and nanoparticle HA activated surfaces and investigated their effect on cell-surface interactions. Previous studies have shown the importance of nanoparticle HA surfaces for improving cell interaction with the implant and so the early stages of bone integration [15,16].

Surface roughness is an important parameter that improves implant osseointegration and bone-to-implant contact [17,18]. As reported by Gehrke et al. [19], the present study also observed a direct relationship between titanium surface roughness and the stimulus for bone formation. As expected, the nanoparticle HA surface showed greater roughness in comparison with the machined surface. Moreover, the chemical composition of the nano group presented a controlled quantity of calcium and phosphorous, which are considered good stimulants/accelerators of osseointegration [20].

To evaluate the early stages of osseointegration on these surfaces, functional tests were performed in vitro using MC3T3-E1, a well-established murine pre-osteoblastic cell line. The live/dead assay found a higher number of viable pre-osteoblastic cells growing on the nanoparticle HA activated surface than on the machined surface. In addition, the initial phase of adhesion and spreading was also investigated by analyzing cell morphology under SEM. In these assays, cells adhered to surfaces in the nano group were characterized by a flatter morphology spreading over the surface, indicating more contact points between cells and surface compared with the mach group. To provide more information about this interaction, focal adhesion formation was analyzed by immunolabeling of vinculin and actin to evaluate the co-localization of these proteins. In agreement with previous results, the cells adhered to nanoparticle HA surfaces displayed more focal contacts than those adhered to mach group surfaces. These findings can be associated with the presence of hydroxyapatite nanocrystals, as the mach group presented an absence of focal adhesion staining.

Previous studies have shown significant increases in bone formation on coated implants in comparison with un-coated implants after 4 weeks healing [9]. These findings indicate that early bone formation is dependent on nanosize hydroxyapatite features but it remains unclear whether this is due to an isolated chemical effect or to nanotopography or a combination of the two [19]. The present results also confirm the importance of testing in vitro bone mineralization directly on the implant surface as a standard practice, which will suggest how bone mineralization will evolve *in vivo and* so help to select biomaterials intelligently to optimize bone integration. Moreover, the results confirm that activated nanoparticle HA and pure crystalline hydroxyapatite provide optimal functional activation and so this surface is a strong candidate as the best means of boosting bone-to-implant integration in clinical practice.

Cytotoxicity analysis is an important parameter for determining whether a given medical device will cause any cell death due to leaching of toxic substances or to direct contact [21]. The live and dead test applied in our study verified both surfaces as non-cytotoxic. The cytotoxic effects of a specific material on a specific cell type can be studied either by directly seeding the cells on the surface of the material or by exposing the cells to the extraction fluid, which is accepted as an indirect toxicity evaluation [22]. The choice of assay will always influence the outcomes of cytotoxicity evaluation of biomaterials [23]. The selection of other parameters, such as cell lines, controls, biochemical assay type and duration of culture are all crucial when evaluating a material’s compatibility [24]. In the present study, osteoblastic cells were selected because clinically the surfaces investigated would be implanted in bone tissue, making the osteoblast cell model entirely appropriate. Moreover, prior to studying tissue response to a material *in vivo*, it is always recommendable to conduct a preliminary in vitro study to obtain some insights into its behavior in the biological environment.

## 5. Conclusions

This study evaluated the cellular response to a nanoparticle HA surface on titanium implants with CaP ions by means of various assays. Biocompatibility was evaluated using MC3T3-E1 cells; no cytotoxicity was observed. Initial cellular attachment showed greater intensity in the nano group in comparison with the machined surface, with a resulting enhancement of osteoblast differentiation in this group. These findings suggest that nanoparticle HA treatment of titanium implants improves mineralization over the surface. However, further clinical studies are necessary to determine the osseointegration response *in vivo*.

## Figures and Tables

**Table 1:** Mean and standard deviation of surface profilometry by group (in µm).Z indicates longest distance recorded between the peak and valley, maximum valley variation; Ra, arithmetic average of the absolute values of all profile points; Rq, the root-mean square of values at all points; Rz, average value of the absolute heights of the 5 highest peaks and the depths of the 5 deepest valleys.

Roughness Parameters	Z	Rq	Ra	Rz
**Mach group**	0.89 ± 0.12	0.14 ± 0.06	0.11 ± 0.04	0.96 ± 0.12
**Nano group**	2.03 ± 0.15	0.54 ± 0.11	0.36 ± 0.09	2.37 ± 0.67
